# Elbow Flexor Adaptations to Failure Versus Repetitions-in-Reserve Training: A Unilateral Within-Participant Study in Resistance-Trained Adults

**DOI:** 10.3390/muscles5030061

**Published:** 2026-08-30

**Authors:** Tiago Vasconcelos, Ana Ruivo Alves, Martin Charles Refalo, Gabriela Chaves Lucas, Pedro Rúben Vasconcelos, João Paulo Brito, Filipe Casanova, José Vilaça-Alves, Rafael Oliveira

**Affiliations:** 1 Research Center in Sports Sciences, Health Sciences and Human Development (CIDESD), 6201-001 Covilhã, Portugal; tiago.vasconcelos@ubi.pt (T.V.); asra@ubi.pt (A.R.A.); jbrito@esdrm.ipsantarem.pt (J.P.B.); josevilaca@utad.pt (J.V.-A.); rafaeloliveira@esdrm.ipsantarem.pt (R.O.); 2 Department of Sport Sciences, University of Beira Interior, 6201-001 Covilhã, Portugal; 3 JPS Health & Fitness, Melbourne, VIC 3042, Australia; mrefalo@deakin.edu.au; 4 University of Trás-os-Montes e Alto Douro, 5001-801 Vila Real, Portugal; gabimaracaba@gmail.com (G.C.L.); pedrovasconcelos2009@live.com.pt (P.R.V.); 5 School of Sport of Rio Maior, Santarém Polytechnic University, 2040-413 Rio Maior, Portugal; 6 Life Quality Research Center (CIEQV), Santarém Polytechnic University, 2001-904 Santarém, Portugal; 7 Research Group in Sports Behavior, Physical Education, and Exercise and Health Sciences (CIDEFES), Lusófona University, 1749-024 Lisboa, Portugal; 8 Center for Studies and Research in Football (NEIF), Lusófona University, 1749-024 Lisboa, Portugal; 9 Research Group in Strength Training and Fitness Activities (GEETFAA), 5001-801 Vila Real, Portugal

**Keywords:** resistance training, hypertrophy, muscle thickness, strength

## Abstract

This study compared elbow flexor hypertrophy and maximal strength adaptations after eight weeks of resistance training performed either to momentary muscular failure or with 1–3 repetitions in reserve (RIR). Nineteen resistance-trained adults completed a unilateral within-participant protocol in which one arm trained to failure and the contralateral arm trained with 1–3 RIR. Participants trained twice weekly using the unilateral preacher curl exercise at 70–80% one-repetition maximum (1RM). Elbow flexor muscle thickness and 1RM strength were assessed before and after the intervention. Bayesian mixed-effects models estimated between-condition differences. Both protocols increased muscle thickness and 1RM strength. The estimated between-condition differences for muscle thickness were 0.032 cm (95% highest density interval [HDI]: −0.044 to 0.113) while the estimated difference in 1RM strength was 0.588 kg (85% HDI: −0.491 to 1.66), with both 95% HDIs spanning zero and including values favoring either protocol. Sex-specific analyses showed the same general patter of responses. Overall, resistance training performed with 1–3 RIR produces similar elbow flexor hypertrophy and maximal strength adaptations to training performed to momentary muscular failures under the loading and volume conditions examined.

## 1. Introduction

Resistance training (RT) is a fundamental modality for eliciting adaptations in skeletal muscle that include hypertrophy of muscle fibers and the enhancement of maximal force-production capacity [1,2,3,4]. Effective RT program designs require the appropriate manipulation of numerous training variables, including the total volume completed [5], the load lifted [4,6,7], the selection of specific exercises, the duration of recovery between sets [8], and the frequency of training stimuli [9], all of which may be influenced by individual factors such as biological sex [10,11], genetics, and lifestyle factors. Among these multifaceted components, the degree of effort exerted within individual RT sets, often operationalized by the proximity to which repetitions are performed relative to the point of momentary muscular failure, has emerged as a variable of considerable scientific interest and practical relevance [12,13,14]. A common method for quantifying this proximity to failure is the use of the repetitions-in-reserve (RIR) scale [15], defined as the estimated number of repetitions remaining before momentary muscular failure is reached, which involves set termination once a target RIR has been achieved. Importantly, a primary gap in the literature assessing proximity to failure is that the RIR associated with each intervention condition is often not directly prescribed, monitored or quantified, which complicates direct comparisons between studies and necessitates post hoc estimations in meta-analyses [16]. Moreover, even when tools like the RIR scale are directly employed, their application is not exempt of limitations, as subjective estimations of RIR have demonstrated small but inherent inaccuracies [12,15,17,18]. Therefore, further investigations into proximity to failure are considered paramount for the development of nuanced, evidence-informed training prescription designed to optimize muscle hypertrophy and strength outcomes [12,13].

Despite the acknowledged importance of manipulating training variables for promoting muscle hypertrophy, debate persists regarding the precise role and necessity of training to, or in close proximity to, momentary muscular failure [12,13,19,20]. Traditionally, training to momentary muscular failure has been advocated as a requisite for ensuring high motor unit recruitment and thereby creating a potent stimulus for adaptation [12,21]. Indeed, a recent series of meta-regressions by Robinson et al. [13] found that muscle hypertrophy increases as RT sets near momentary muscular failure. The interpretation of this finding, however, is complicated by notable limitations, namely, that the RIR values analyzed were estimated by the researchers post hoc and that the analysis combined data from studies with considerable methodological heterogeneity. These collective limitations underscore the findings from Refalo and colleagues [12], whereby a robust within-subject design with direct (and highly accurate) RIR prediction revealed equivalent hypertrophic outcomes between training to momentary muscular failure and 1 to 2 RIR. Importantly, in the present study, RIR was prospectively prescribed as part of the training intervention rather than estimated retrospectively, with participants terminating sets at a target of 2 RIR within an acceptable range of 1–3 RIR following dedicated familiarization and under direct supervision throughout the intervention.

For strength development, Robinson et al. [13] conversely reported a negligible relationship between proximity to failure and strength gains, suggesting that the influence of this proximity to failure is likely outcome-specific. This potential dichotomy, whereby proximity to failure may be a more critical determinant of muscle hypertrophy than for strength, has significant implications for program design, indicating that training prescriptions may need to be tailored to the specific adaptation goal.

Furthermore, while biological sex is often considered a variable influencing training adaptations, recent evidence suggests that apparent sex differences may be underpinned by disparities in baseline strength rather than sex per se [22,23]. For instance, when participants are matched for relative strength, differences in muscle activation patterns [24] and rapid force production characteristics [23] between males and females are largely attenuated. This suggests that baseline relative strength may account for some apparent sex-related differences in neuromuscular characteristics and therefore should be considered when interpreting comparisons between males and females. Consequently, it is plausible that the physiological response to manipulating proximity to failure is comparable between sexes provided that the training status and relative intensity are appropriately controlled [25,26].

Although previous within-participant research has directly compared momentary muscular failure with prospectively prescribed RIR-based training, this work has primarily characterized hypertrophic responses of the quadriceps and did not specifically examine protocol responses separately in males and females [27]. Whether comparable responses extend to other muscle groups, particularly the elbow flexors, and to exercise-specific maximal strength adaptations therefore remains less well characterized. Moreover, sex-stratified estimates of the response to different proximities to failure remain scarce [10,11,25,26,28]. Accordingly, extending this experimental approach to upper-limb musculature while providing exploratory estimates of the FAIL-versus-RIR contrast separately in males and females may help inform the generalizability of previous findings and the design of future adequately powered investigations.

Therefore, the primary purpose of this study was to compare elbow flexor hypertrophy and maximal strength adaptations following eight weeks of resistance training performed either to momentary muscular failure or with a target of 1–3 repetitions in reserve, using a unilateral within-participant design in resistance-trained men and women. Given the limited evidence directly examining whether adaptations to RT proximity to failure differ between sexes, sex-stratified analyses were also conducted as exploratory, hypothesis-generating analyses to characterize the observed response patterns in males and females. We hypothesized that muscle hypertrophy and maximal strength outcomes will be similar between the RT protocols for the whole sample and for males and females separately.

## 2. Results

### 2.1. Change in Elbow Flexor Thickness

**Figure 1:**
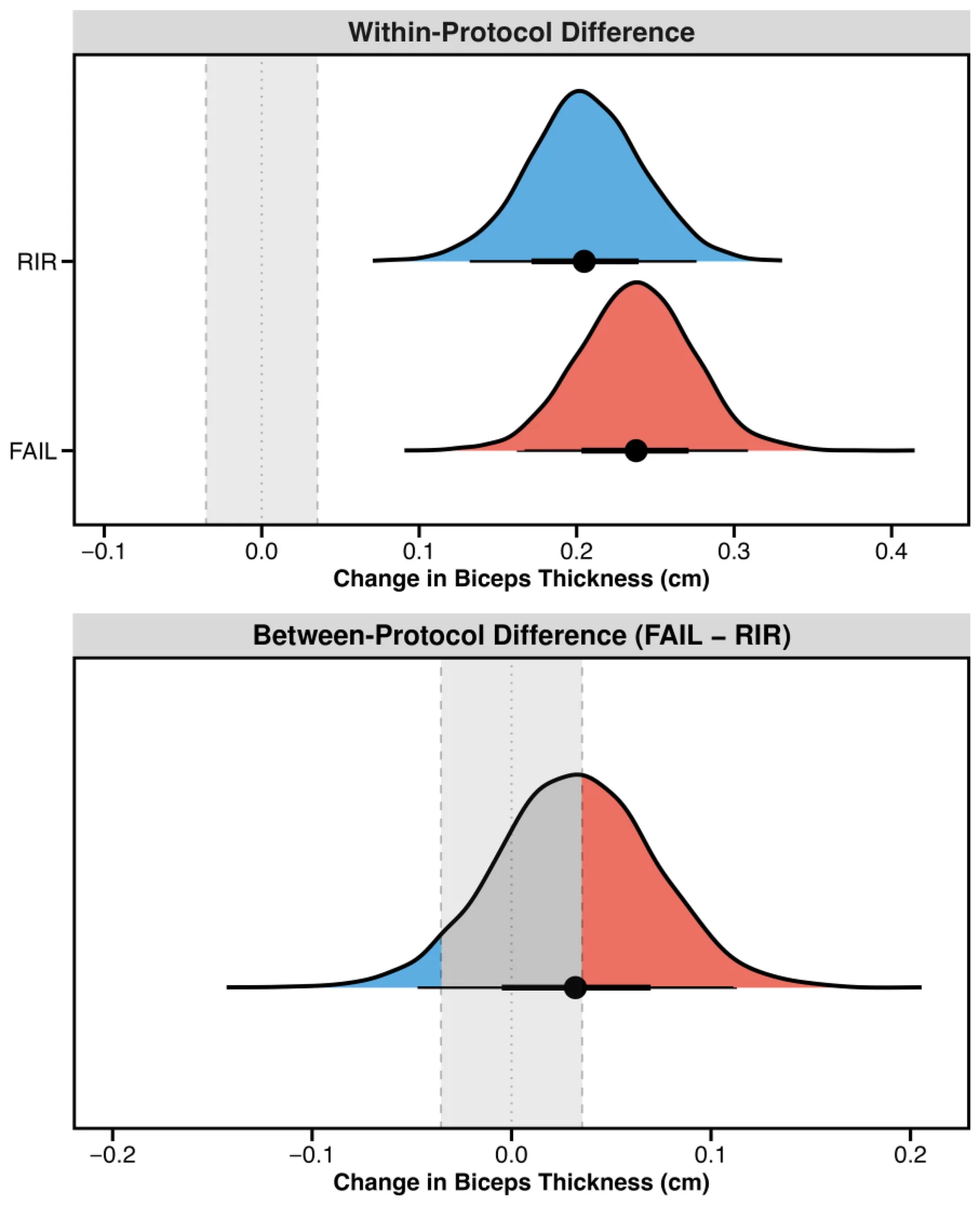
Posterior distribution for within- and between-protocol changes in elbow flexor thickness. Elbow flexor thickness was calculated as the average of three sites along the muscle belly. The plots display the posterior distributions, which show the central tendency (point estimate = mean) and the 95% highest density credible interval (HDI; thick black lines). The vertical shaded grey represents the region of practical equivalence (ROPE). In the “within-protocol difference” plots, blue distribution represents the RIR protocol and red distributions represent FAIL protocol. In the “between-protocol difference” plots, negative estimates (blue area) favor the RIR protocol, while positive estimates (red area) favor the FAIL protocol.

Raw elbow flexor (50%, 60% and 70% combined) thickness at pre- and post-intervention is displayed in Supplementary Figure S3. Elbow flexor thickness was estimated for RIR [0.205 cm (HDI: 0.134 to 0.277); pd = 100%] and FAIL [0.237 cm (HDI: 0.167 to 0.308); pd = 100%] from pre- to post-intervention. Probability of change in elbow flexor thickness above the ROPE was also estimated for RIR (pd > ROPE = 100%) and FAIL (pd > ROPE = 100%). The between-protocol estimate was positive [0.032 cm (HDI: −0.044 to 0.113); pd = 80%]. The pd indicated an 80% posterior probability that the difference favors FAIL; however, the 95% HDI included values in both directions, and only 47% of the posterior distribution exceeded the ROPE, indicating that the between-protocol difference was small and uncertain, as displayed in Figure 1.

### 2.2. Change in Elbow Flexor Thickness for Males and Females Separately

Bayesian estimates for changes in elbow flexors thickness for males and females (within- and between-protocol), separately, are presented in Table 1. Exploratory sex-stratified estimates showed increases in muscle thickness under both protocols in males and females. Between-protocol differences were small and uncertain in both males [0.035 cm (95% HDI: −0.075 to 0.136)] and females [0.027 cm (95% HDI: −0.095 to 0.146)], with the HDIs including values in both directions. Given the limited subgroup sample sizes, these sex-stratified estimates should be considered exploratory and hypothesis-generating and should not be interpreted as confirmatory evidence of equivalence between protocols within either sex. Posterior distributions for these estimates are displayed in Supplementary Materials (Figure S4).

### 2.3. Analysis of Standardized Effect Estimates

**Figure 2:**
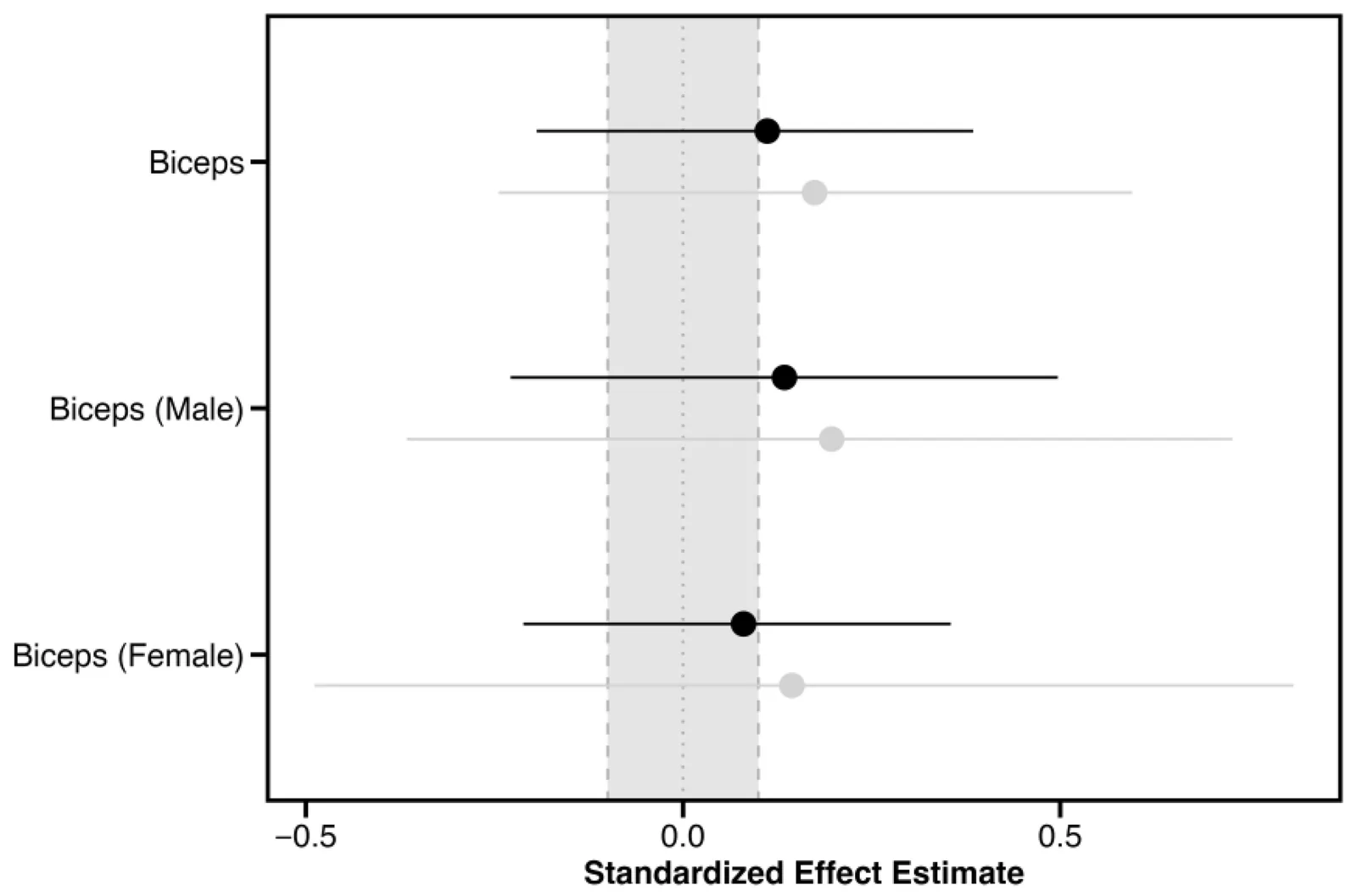
Standardized effect estimates for between-protocol differences in muscle thickness. The figure displays the standardized effect estimates (point estimates = dots) and their corresponding 95% highest density credible intervals (HDIs) for the between-protocol difference (FAIL vs. RIR). Estimates are shown for the entire cohort (males and females) and for males and females separately. The solid black dot points represent the estimates derived using weakly informative priors, while the solid grey points represent the estimates derived using default (non-informative) priors. The vertical shaded grey area represents the region of practical equivalence (ROPE). Negative estimates favor the RIR protocol, while positive estimates favor the FAIL protocol.

Hedges’ g effect estimates for male and female elbow flexor thickness (overall and separate) can be found in Table 2 and displayed in Figure 2. Using weakly informative priors, the standardized between-protocol point estimates were small for the full cohort (Hedges’ g = 0.112; 95% HDI: −0.194 to 0.385), males (g = 0.134; −0.229 to 0.497), and females (g = 0.080; −0.212 to 0.355). In all cases, the HDIs included effects in both directions, indicating substantial uncertainty regarding the between-protocol differences.

### 2.4. Change in Repetition Maximum Strength

**Figure 3:**
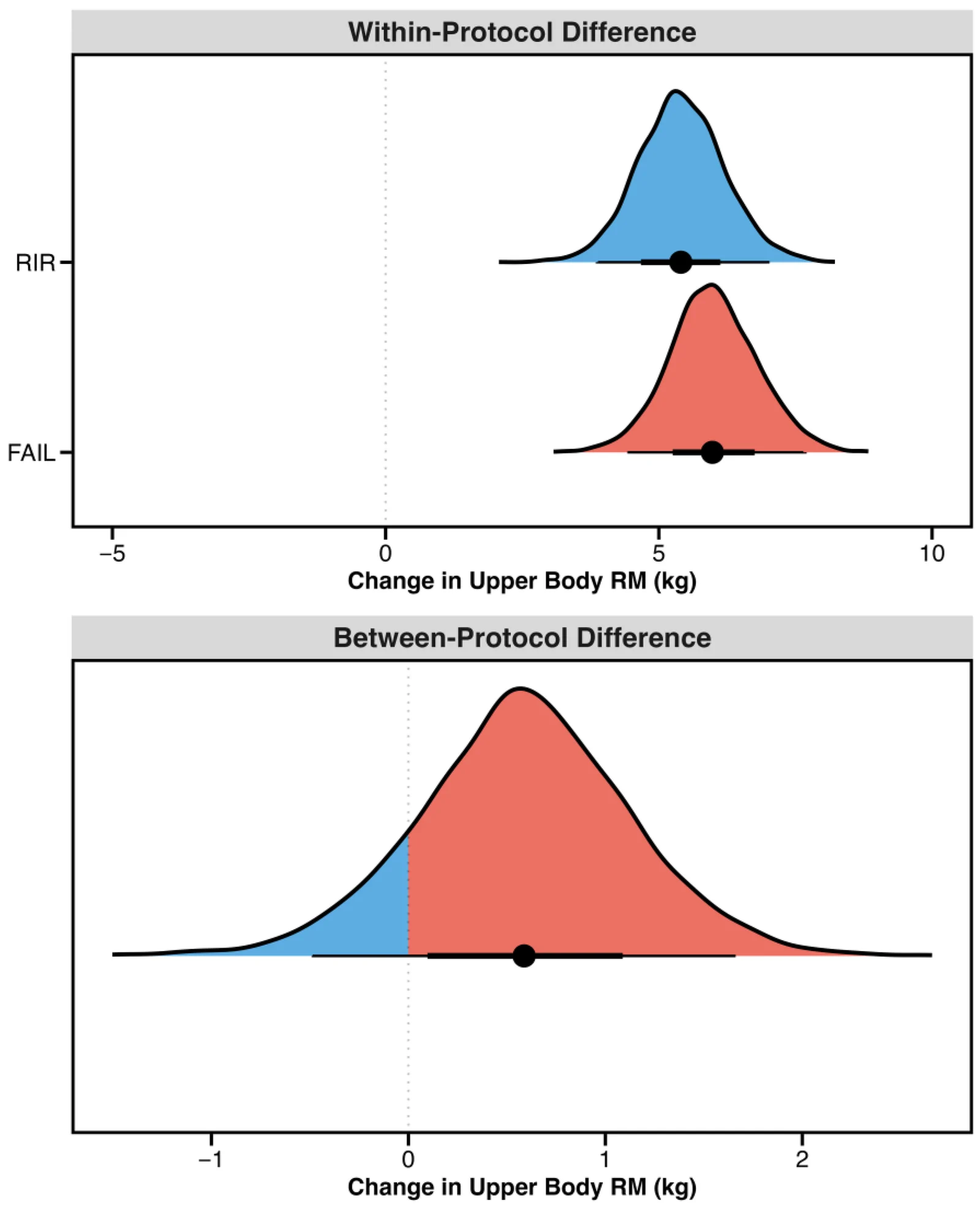
Posterior distributions for within- and between-protocol changes in 1-repetition maximum (1RM) strength. Upper body RM refers to the unilateral preacher curl 1RM. The plots display the posterior distributions, which show the central tendency (point estimate = mean) and the 95% highest density credible intervals (HDI; thick black lines). In the “within-protocol difference” plots, blue distributions represent the RIR protocol and red distributions represent the FAIL protocol. In the “between-protocol difference” plots, positive estimates (red area) favor the FAIL protocol, while negative estimates (blue area) favor the RIR protocol.

Raw elbow flexor maximum strength was estimated to increase for RIR [5.41 kg (HDI: 3.86 to 6.97); pd = 100%] and FAIL [6.00 kg (HDI: 4.37 to 7.58); pd = 100%] from pre- to post-intervention. The between-protocol estimate was positive [0.588 kg (HDI: −0.491 to 1.66); pd = 87%]; the pd indicates an 87% posterior probability that the difference favored FAIL; however, the 95% HDI included values in both directions, indicating that the between-protocol difference remained small and uncertain. Posterior distributions for within- and between-protocol differences are displayed in Figure 3.

### 2.5. Change in Elbow Flexor Maximal Strength for Males and Females Separately

Bayesian estimates for changes in elbow flexor maximal strength for males and females (within- and between-protocol), separately, are presented in Table 3. Sex-stratified estimates showed increases in 1RM strength under both protocols in males and females. The between-protocol difference was 0.835 kg (95% HDI: −0.661 to 2.20; pd = 88%) in males and 0.248 kg (95% HDI: −1.308 to 1.97; pd = 62%) in females, with both HDIs including values in either direction. Posterior distributions for these estimates are displayed in Supplementary Materials (Figures S5 and S6).

## 3. Discussion

The principal finding of this investigation was that in trained males and females, RT sets with a 1 to 3 RIR may promote similar muscle hypertrophy and strength development as training to momentary muscular failure. Importantly, this pattern of comparable outcomes between the protocols was consistent across both male and female participants. Unlike many previous research studies that do not involve RIR quantification in the non-failure protocol [16], these findings provide a direct experimental comparison of RT performed with RIR (involving intra-set RIR prediction) versus to momentary muscular failure in elbow flexors in a mixed-sex cohort, strengthening the evidence base in this area.

Our primary analysis indicated a small estimated difference in muscle hypertrophy between FAIL and RIR protocols, with less than 50% of the posterior distribution exceeding the predefined ROPE for the elbow flexors. Thus, the posterior estimates were more compatible with a small or practically trivial between-protocol effect than with a clear advantage for either condition. However, the uncertainty surrounding the estimate precludes interpreting these findings as definitive evidence of equivalence or as demonstrating the absence of a meaningful difference. Within the present design, the results therefore suggest that reaching momentary muscular failure over terminating sets with 1–3 RIR was likely small, within the present applied context. Thus, training in close proximity to failure appeared to be enough to produce similar hypertrophy between conditions in this design. Nonetheless, recent meta-analysis shows that muscle hypertrophy steadily increases as sets are terminated closer to momentary muscular failure (on average) [13]. Larger-scale analyses treating RIR as a continuous variable may indeed detect smaller differences in muscle hypertrophy between RIR conditions; however, the true accuracy of post hoc RIR estimations required for meta-analysis is unclear. Therefore, whether a true linear relationship between proximity to failure and muscle hypertrophy exists across multiple sets is still in question, with more studies quantifying RIR effectively in non-failure groups required for meta-analysis. Moreover, our analyses revealed a consistent pattern of outcomes across both sexes: for both male and female participants, the FAIL and RIR protocols were similarly effective for promoting gains in muscle hypertrophy. Indeed, a recent meta-analysis found that females have a similar potential to induce muscle hypertrophy as males, particularly when considering relative increases in muscle size from baseline [26]. Overall, the present findings provide further evidence that reaching close proximities to failure (1–3 RIR) can provide comparable muscle hypertrophy to momentary muscular failure across both trained men and women.

It is possible for hypertrophic responses to differ across muscle groups, with possible muscle-specific trends emerging when examining the elbow flexors independently in the present study. For the elbow flexors, our secondary analysis of standardized effect estimates (using weakly informative priors) showed a low but favorable (>50%) probability that the FAIL protocol elicited a slightly greater hypertrophic response versus RIR that exceeded the ROPE. However, the favorability of FAIL over RIR for elbow flexor thickness was less probable, and therefore considered negligible, when analyzing raw units. This observation for the elbow flexors aligns with Martorelli et al. [29], who observed no additional benefits for muscle thickness in young women when training to failure compared to a volume-equated non-failure protocol. Similarly, other studies investigating the elbow flexors have reported similar hypertrophic outcomes between failure and non-failure conditions [30,31]. Inconsistencies in training status, volume control, and program design likely contribute to the divergent findings and highlight that the hypertrophic response is context dependent.

Therefore, while speculative, it is plausible that certain anatomical or physiological characteristics of specific muscle groups might make them particularly responsive to a certain proximity to failure across a whole workout.

For the outcome of maximal strength, the present investigation found that both the FAIL and RIR protocols were equally effective for enhancing 1RM performance. Furthermore, this general finding held true when analyzed by sex, as no clear or consistent advantage for either protocol emerged for males or females on the preacher curl. The most plausible mechanism for this similarity is that the high load itself was the primary driver of strength adaptations, as demonstrated in multiple meta-analyses [32]. Given that both the FAIL and RIR protocols in the present study employed the same high loads (e.g., 70–80% 1RM), it is likely that the load provided sufficient stimulus to drive neural and muscular adaptations, rendering the condition of training to momentary muscular failure redundant for this outcome. Indeed, recent meta-regression results show that terminating RT sets closer to momentary muscular failure does not positively impact maximal strength development [13]. This flexibility in the influence of proximity to failure on RT outcomes is particularly relevant for strength-focused training, as high levels of neuromuscular fatigue can impair neural drive and reduce the capacity to produce maximal force within a training session [33]. Over a training block, this repeated attenuation of force production could theoretically blunt the cumulative stimulus for long-term strength adaptations; however, this is yet to be demonstrated in the literature. Overall, the findings of the present study show that RT can be effectively performed with a 1–3 RIR target for strength development, possibly allowing for better fatigue management than reaching momentary muscular failure. From a practical perspective, prescribing 1–3 RIR may provide a viable alternative to systematically training to failure when similar hypertrophy and strength adaptations are desired while managing fatigue. Because training to failure has also been associated with greater perceived discomfort and exertion [34], avoiding systematic failure may have potential implications for adherence; however, adherence was not directly assessed in the present study.

The present investigation possesses several methodological strengths that enhance confidence in its findings. A primary strength is the use of a unilateral, within-subject design, which minimizes the influence of inter-individual variability and increases statistical power for detecting changes. Furthermore, the present study employed direct RIR prediction following a dedicated familiarization period, providing a more direct and ecologically valid comparison between the training protocols. The inclusion of both males and females also broadens the generalizability of the findings.

Nonetheless, these strengths are accompanied by limitations that provide clear avenues for future research. These findings reflect adaptations within the context of a controlled single-joint, single-exercise training model and should not be extrapolated to higher-volume, multi-exercise or whole-body resistance training, in which the cumulative fatigue associated with repeated sets and exercises may influence responses to training to momentary muscular failure. Furthermore, the present findings are specific to young, resistance-trained adults and should not be generalized to untrained individuals, older adults, or clinical populations without further investigation. Importantly, background training exposure was equivalent between limbs within each participant, minimizing differential bias between conditions. The sample size, while consistent with similar research, was primarily justified by pragmatic considerations and may not have been sufficient to reliably detect very small between-protocol differences. Although participants were allowed to maintain their habitual resistance-training routines for non-targeted muscle groups, the unilateral within-participant design minimized potential confounding effects of background training exposure. Any additional upper-body training performed outside the study would have affected both limbs similarly, thereby preserving the internal validity of the between-protocol comparison. Nevertheless, future studies may further standardize external training exposure to strengthen causal inference.

Furthermore, we acknowledge that the within-subject design introduces the potential for cross-education effects, particularly for strength outcomes. While cross-education is most clearly demonstrated when one limb remains untrained, we acknowledge that some degree of neural transfer cannot be fully excluded even when both limbs are trained. Therefore, the internal validity of the strength outcomes may be partially compromised, and strength-related findings should be interpreted with caution. Additionally, limb dominance was not considered during condition allocation, and given the relatively small sample, a potential influence of limb dominance on strength outcomes cannot be fully excluded.

Although participants were classified as resistance-trained, the intervention focused exclusively on a single joint, which may limit ecological validity compared to multi-joint training programs typically employed by this population. Additionally, the findings are specific to the single-joint exercise employed; therefore, future research should replicate this comparison using multi-joint, free-weight exercises where the technical and neurological demands differ. Future research with larger cohorts, varied exercises and muscle groups, and broader training contexts will help clarify the nuanced role of proximity to failure in driving long-term adaptations.

## 4. Materials and Methods

### 4.1. Experimental Approach to the Problem

A unilateral, within-subject randomized experimental design was utilized to compare the effects of an 8-week RT intervention on muscular adaptations, specifically muscle hypertrophy and maximal strength of the elbow flexors. The intervention consisted of two supervised training sessions per week, during which participants performed identical training volume between limbs for preacher curls. Following completion of all baseline (PRE) assessments, one limb was randomly assigned (coin toss) to a protocol training to momentary muscular failure (FAIL) and the contralateral limb assigned to a protocol training with repetitions in reserve (RIR). Thus, no limb-specific protocol allocation had been made at the time of baseline testing. Limb dominance was not considered in the randomization process. This design ensured that each participant served as their own control [27]. The primary outcomes of muscle hypertrophy and maximal strength were assessed via ultrasound imaging and 1-repetition maximum (1RM) testing, respectively. These assessments were conducted at baseline (PRE) and following the intervention (POST), with the RIR familiarization completed only at PRE. Because randomization occurred only after completion of PRE assessments, the investigator performing baseline testing had no knowledge of any subsequent limb-specific protocol allocation. Investigator conduction the POST 1-repetition maximum and ultrasound assessments remained blinded to limb-specific protocol allocation.

### 4.2. Participants

Initially, twenty-three resistance-trained young adults (13 males and 10 females) were recruited for this experiment. Prior to the commencement of the intervention, one female participant was excluded due to an inability to complete all baseline ultrasound measurements. Consequently, twenty-two participants (13 males and 9 females) began the 8-week training intervention. During the experimental period, three participants (two males and one female) were further excluded from the final analysis. The reasons for exclusion were failure to meet the minimum required training session attendance (n = 2, males) and an unrelated injury sustained mid-experiment (n = 1, female).

**Figure 4:**
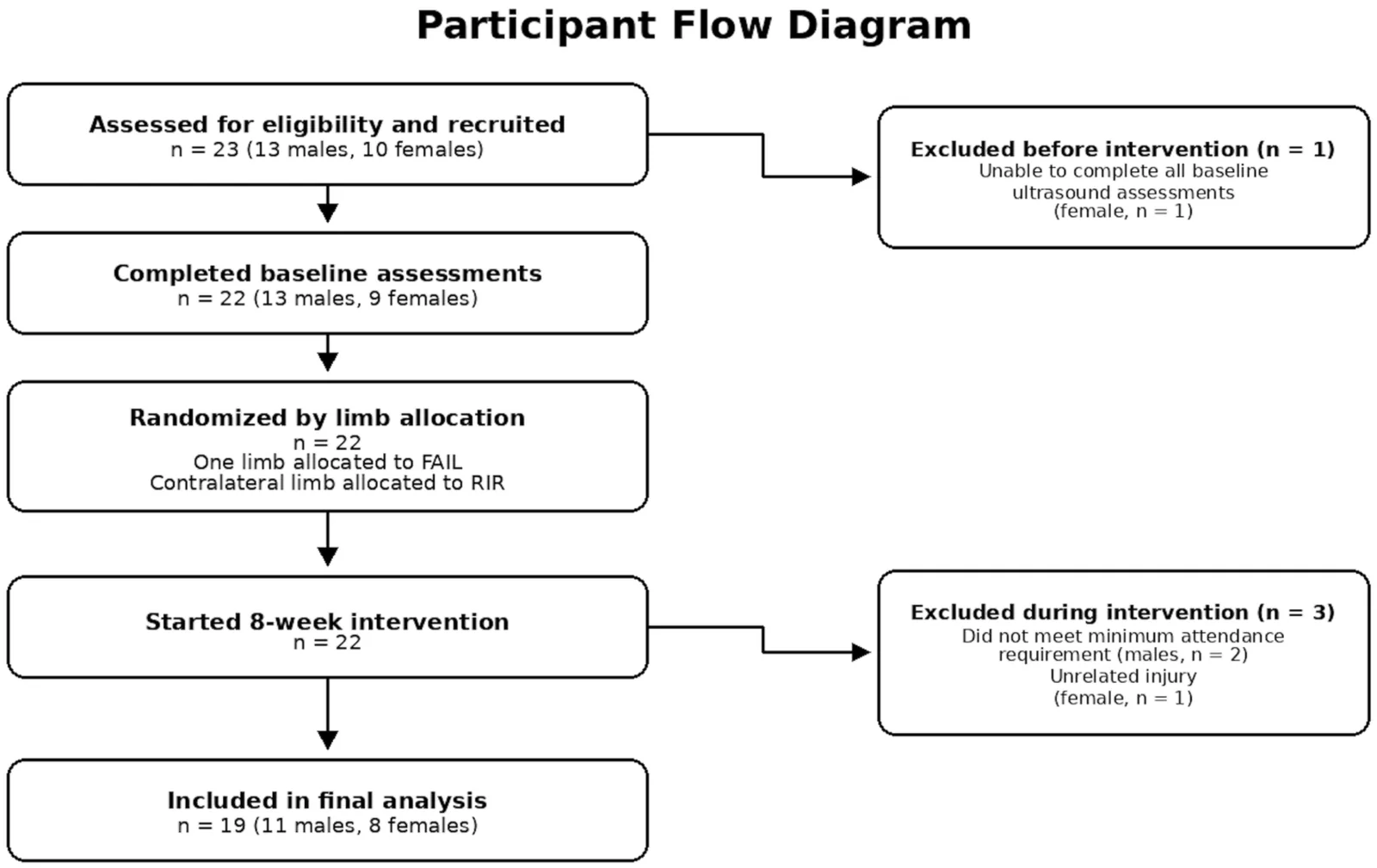
Participant flow through the study. Twenty-three resistance-trained adults were recruited. One female participant was excluded before the intervention because she was unable to complete all baseline ultrasound assessments. Twenty-two participants completed baseline assessments and subsequently underwent limb allocation. Three participants were excluded during the intervention. The final analytic sample comprised 19 participants (11 males and 8 females). FAIL, momentary muscular failure; RIR, repetitions in reserve.

Thus, the final analysis was conducted on a sample of nineteen participants, comprising eleven males (mean ± SD: age = 20.4 ± 1.8 years; height = 172.7 ± 5.6 cm; body mass = 74.9 ± 7.6 kg) and eight adult females (mean ± SD: age = 24.8 ± 4.7 years; height = 164.1 ± 8.0 cm; body mass = 63.0 ± 10.1 kg). Participant recruitment, exclusions, intervention participation, and the final analytic sample are summarized in Figure 4.

Participants were recruited from the local University School of Sport via flyers, email announcements, and word of mouth. To be eligible for inclusion in the study, participants were required to be: (1) currently engaged in regular RT for a minimum of two years, consistently at least three times per week [8,27]; (2) free from any existing musculoskeletal injuries or medical conditions that would contraindicate participation in a demanding RT program or affect their ability to perform the required exercise and testing procedures; (3) have abstained from the use of anabolic steroids at any point in their lifetime; and (4) not currently participating in any additional training program specifically targeting the elbow flexors outside their habitual resistance training routines. Pre-existing ergogenic supplementation was permitted provided that the supplementation regimen remained unchanged throughout the intervention; however, no participant reported ergogenic supplement use at baseline or during the study.

Furthermore, data from participants who commenced the intervention were excluded from the final analysis if they failed to adhere to the predetermined minimum attendance requirement of 85% of the scheduled supervised training sessions, commenced any new ergogenic supplementation during the experimental period contrary to initial instructions, or sustained an injury, either related or unrelated to the study protocol, that precluded their continued participation in the training intervention or the completion of all post-intervention assessment procedures.

Participants provided written informed consent before all study procedures, potential risks, and benefits were fully explained. The study was approved by the Research Ethics Committee from the same university (Ethics Approval Number: 19-2023ESDRM) and conducted in accordance with the Declaration of Helsinki.

### 4.3. Sample Size Justification

The target sample size for this study was determined a priori. Aiming to retain a minimum of 19 participants for the final analysis after accounting for potential attrition, a total of 23 resistance-trained young adults were recruited. This sample size was primarily justified based on pragmatic considerations and resource constraints [35], which are common in supervised 8-week RT interventions that require significant time, personnel, and financial resources [27,36]. Furthermore, this sample size is consistent with or greater than that of similar within-subject studies investigating the effects of proximity to failure on muscular adaptations [27,36,37]. This sample size was further evaluated using a sensitivity power analysis (G*Power, Version 3.1.9.7) focused on the primary paired comparison between the FAIL and RIR conditions. With the final sample of 19 participants, a two-sided paired-sample test with alpha level (α) = 0.05 and a power (1 − β) of 0.80 yielded a minimum detectable standardized between-protocol effect of approximately Cohen’s dz = 0.68. Accordingly, the study had limited sensitivity to reliably detect small or modest between-protocol effects [37]. This sensitivity analysis is provided to characterize the informational constraints imposed by the final sample rather than as the basis for the primary Bayesian inference. A Bayesian statistical framework was selected to directly estimate the posterior distribution of plausible effect sizes and quantify the uncertainty surrounding the between-protocol estimates [38]. This approach facilitates interpretation of the magnitude and precision of the observed effects without relying on binary null-hypothesis significance testing [39].

### 4.4. Procedures

Participants completed an 8-week supervised RT intervention, attending sessions twice per week. Each session was separated by a minimum of 48 h and a maximum of 96 h to allow adequate recovery [9,14,21,27]. All training sessions were directly supervised by an investigator to ensure adherence to the assigned protocols and proper exercise technique. The intervention targeted unilateral preacher curls (for elbow flexors) performed on the limb assigned to the FAIL protocol and the limb assigned to the RIR protocol. The exercise was performed seated on a preacher curl bench with the upper arm supported against the pad, moving the dumbbell through the standardized range of motion established during familiarization. For the limb assigned to the FAIL protocol, participants performed all sets to momentary muscular failure. Momentary muscular failure was defined as the inability to complete the concentric portion of the current repetition despite attempting to do so (for at least two seconds) and with the prescribed technique [14,27,40,41]. For the limb assigned to the RIR protocol, participants terminated sets based on a perceived RIR target. Specifically, they aimed for a target of 2 RIR within an acceptable range of 1–3 RIR for each set [14,27,42,43]. Participant-reported RIR at set termination was recorded at the set level throughout the supervised intervention. To ensure continued adaptation and apply progressive overload, the total number of sets performed per exercise was increased midway through the intervention [5,27,44,45], as detailed in Table 4.

The initial training load was selected to correspond to approximately 70–80% of the participant’s PRE 1RM, with the goal of performing 8–12 repetition at the prescribed FAIL or RIR endpoint [3,4,6,27]. Progressive overload was implemented throughout the intervention; if a participant successfully completed 12 or more repetitions in a set, the load was increased for the subsequent training session by approximately 1–2 kg [3,27,46]. Furthermore, intra-session load adjustments were made if repetition performance consistently fell outside the target 8–12 repetition range while adhering to the specified FAIL or RIR endpoint. Specifically, for the FAIL condition, if a participant was unable to achieve a minimum of 8 repetitions with the selected load on a given set, the load was reduced (by approximately 1–2 kg) for the subsequent set; however, to avoid excessive load manipulation, some decay in repetitions (e.g., down to a minimum of approximately 4–5 repetitions per set) was permitted before such a load reduction was implemented. For the RIR condition, if a participant consistently performed fewer than 8 repetitions to achieve 2 RIR with the selected load, the load was similarly reduced for the subsequent set. Minor deviations from 8 to 12 repetitions target (e.g., achieving 7 repetitions at 2 RIR) were tolerated on occasion to prevent excessive intra-session load adjustments, with the participant’s self-regulation of repetitions to maintain 2 RIR being the primary mechanism for achieving the target within the given load.

Rest intervals were standardized at 2 min between sets. Sets were alternated between limbs throughout each session; thus, each set performed with one limb was followed by a set with the contralateral limb. To balance potential order effects between conditions, the limb initiating the exercise (FAIL vs. RIR) was alternated between training sessions. Participants were permitted to maintain their habitual resistance training routines for non-targeted muscle groups to preserve ecological validity and facilitate adherence to the intervention. Adherence to this instruction was verbally confirmed at each supervised session, and no deviations were reported. Detailed information regarding permitted and restricted exercises during the intervention period is provided in the Supplementary Materials (Section S1.1).

### 4.5. RIR Scale Familiarization

Prior to baseline data collection, all participants completed a dedicated familiarization period focused on calibrating their perception of the RIR scale. These sessions were separated by at least 48 h. During these familiarization weeks, participants performed unilateral preacher curls bilaterally. Throughout this process, participants had continuous visual access to a printed RIR scale displayed prominently in their view. Then, they were asked the following question: “Do you feel you could do exactly 2 more repetitions?” Upon reaching the target perception, participants would signal verbally, and the investigator would instruct them to continue to failure to verify if the actual repetitions performed matched the predicted RIR, allowing for immediate calibration of their perception. This procedure, conducted under supervision, aimed to enhance their ability to accurately gauge RIR during the subsequent training intervention, consistent with the familiarization approach utilized in similar research investigating RIR-based training [27,42]. A visual demonstration of the unilateral preacher curl technique and standardized range of motion is provided in the Supplementary Materials (Section S1.2).

### 4.6. Muscle Thickness Assessment

Muscle thickness of the elbow flexors (biceps brachii and brachialis), for both the intervention limb (FAIL or RIR protocol) and the contralateral limb was assessed PRE and POST the 8-week intervention period. All measurements were performed using B-mode ultrasound imaging (A6, B&W, Shenzen, China) equipped with a multifrequency linear-array transducer probe (L45, Sonoscope, Shenzen, China), operating at a frequency of 7.5 MHz and with an image acquisition width of 50 mm. Participants were instructed to abstain from any strenuous exercise for at least 72 h prior to each assessment sessions (PRE and POST). Upon arrival at the laboratory, participants rested in a supine position for a minimum of 10 min before any measurements were taken to allow for body fluid stabilization. Representative ultrasound images and additional reliability details are presented in the Supplementary Materials (Section S1.3).

Measurement sites were precisely identified and marked with an indelible surgical marker. For the biceps brachii (BB), with the participant supine and the arm positioned alongside the body, fully relaxed, with the elbow fully extended, and the forearm supinated, sites were marked with an indelible surgical marker at 50%, 60% and 70% of the distance from the acromion process to the cubital fossa [47]. The muscle thickness values from these three sites were then averaged to obtain a single BB muscle thickness value for each limb. To ensure accurate relocation for POST assessments, all measured distances from anatomical landmarks to the precise measurement sites were recorded during the PRE assessment to minimize inter-session error. A generous amount of water-soluble transmission gel was applied for acoustic coupling. The ultrasound transducer was oriented perpendicular to the muscle tissue interface, ensuring minimal pressure to avoid tissue compression. At each site, a minimum of two images were captured and saved digitally. If the muscle thickness values from these initial two images differed by more than 0.5 mm, a third image was acquired.

Image settings were optimized and maintained consistently throughout all assessments. Muscle thickness for BB was defined as the linear distance between the superficial fascia adjacent to the subcutaneous fat layer and the deep interface overlying the bone.

Image analysis was performed using on-screen digital calipers of the ultrasound system. The average muscle thickness from the selected images (either two images if their initial difference was ≤0.5mm or three images if a third was required) for each site was used for statistical analysis. Muscle thickness assessment sessions were always conducted after a minimum of 72 h without training or engaging in strenuous physical activity. Ultrasound image acquisition was performed by two experienced investigators, whereas all image analyses were conducted by a third investigator. Image files were coded so that the analysis was blinded to participant identity, and limb specificity was blinded to the limb’s training protocol allocation (FAIL or RIR) and, where feasible, to the assessment time-point (PRE and POST). The typical error (TE) and intraclass correlation (ICC) from pre- and post-testing ultrasound assessments are TE = 0.035 and ICC = 0.997 for the elbow flexors (combination of all sites).

### 4.7. Maximal Strength Assessment (1RM Testing)

Maximal strength for the elbow flexor was assessed via a one-repetition maximum (1RM) test for each limb PRE and POST the 8-week intervention period. A single testing session was utilized to determine 1RM at PRE, and another single session at POST. A specific warmup set of 8–10 repetitions was performed using a light load, estimated to be approximately 40–50% of the perceived 1RM. After a 2–3 min rest period, a second warmup set of 3–5 repetitions was performed at a moderate load, estimated to be approximately 60–70% of the perceived 1RM. Following a 3–5 min rest period, the first 1RM attempt was made. If the attempt was successful, the load was incrementally increased by approximately 1–2 kg for the unilateral preacher curl, based on the participant’s perceived exertion and the investigator’s judgment. After a 3–5 min rest interval, another attempt was made. This process was repeated until the participant failed to complete a repetition with correct technique [48]. The 1RM was recorded as the heaviest load successfully lifted for a single repetition with acceptable form, typically achieved up to 4 maximal attempts.

All 1RM tests, as well as the training intervention, were performed using commercial-grade equipment (Technogym, Gambettola, Italy), including a preacher curl bench with adjustable dumbbells. The same investigator conducted all 1RM testing sessions (PRE and POST). Standardized verbal instructions were provided before each lift, and consistent verbal encouragement was given during all 1RM attempts to promote maximal effort. The investigator remained blinded to the specific training protocol (FAIL or RIR) assigned to each limb.

### 4.8. Statistical Analyses

All statistical analyses were conducted using R (v. 4.4.1 [49]) within a Bayesian framework. Bayesian linear mixed-effects models were fitted using the brms package (v. 2.23.0) [50,51,52]. Posterior draws were extracted using tidybayes (v. 3.0.7) [53], and estimated marginal effects were calculated using emmeans (v. 1.10.6) [54]; the probability (i.e., percentage value ranging from 0% to 100%) that an estimate was in favor of a given protocol was calculated manually by examining the proportion of posterior draws that met the criteria of interest (e.g., >0) and denoted as the probability of direction (pd). Both outcomes were analyzed as PRE-to-POST change scores. For muscle thickness, fixed effects included protocol, sex, the protocol × sex interaction, and measurement site, with participant-specific random intercepts and protocol slopes and a random intercept for limb nested within participant; the standardized model used the same structure. For maximal strength, fixed effects included protocol, sex, and the protocol × sex interaction, with a random intercept for participant. For muscle thickness outcomes, we also calculated the probability that a certain change in muscle thickness exceeded the TE, which was used to define the “region of practical equivalence” (ROPE) and denoted it as “pd > ROPE”. Non-informative priors (i.e., default “brms” priors) were used for all model parameters across all outcome measures in raw units. However, we also expressed individual changes in muscle thickness in standardized units, by calculating z-scored ESs using a residual-based standardization approach. Raw muscle thickness change scores were first computed and then divided by the residual standard deviation (SD) from a linear model predicting muscle thickness change from protocol, sex, and their interaction. This residual SD reflects the unexplained variation in muscle thickness change and serves as a stable, protocol-independent denominator. As per recommendations by Penney et al. [55], the resulting standardized effect estimates are interpretable as standardized effect sizes (ESs) and are preferred over traditional pooled SD methods when dealing with unequal variances or unbalanced designs. These standardized mean differences were converted to Hedges’ g by applying the small-sample correction factor (J). Standardized effect estimates were restricted to muscle thickness as a secondary analysis to contextualize hypertrophic effects relative to the standardized ROPE; maximal-strength changes were retained in kilograms because all participants completed the same 1RM test, providing a directly interpretable absolute scale. For models analyzing standardized effect estimates, weakly informative priors (informed by previous data [27]) were used for “protocol” and “sex” parameters. The ROPE for standardized effect estimates was defined as −0.1 to 0.1, corresponding to half of the conventional threshold for a small standardized effect (0.2) and representing a pragmatic range of effects considered practically negligible [39]. This conventional threshold was applied only to the secondary standardized analyses and has also been used in previous resistance training research [56,57]. Inferences from all analyses were made from posterior samples generated using the Hamiltonian Markov chain Monte Carlo method and via the use of high-density credible intervals (HDIs). Inferential priority was given to our primary analyses of raw units, followed by our secondary analyses of standardized effect estimates, of which priority was given to models involving weakly informative priors versus non-informative priors. Model convergence and sampling quality were assessed via standard diagnostic checks (e.g., R-hat statistics ≤ 1.01, visual inspection of trace plots, effective sample sizes, and posterior predictive checks). All raw data of outcome measures (in text and figures) are presented as mean and standard deviation. Test–retest reliability of ultrasound-derived muscle thickness measures was assessed using intraclass correlation coefficients (ICC; two-way mixed-effects model, absolute agreement) and typical error (TE). ICC values were interpreted as poor (<0.50), moderate (0.50–0.75), good (0.75–0.90), and excellent (>0.90) [34]. Ultrasound test–retest reliability was excellent for elbow flexor muscle thickness (ICC = 0.997; TE = 0.035 cm).

## 5. Conclusions

This study demonstrates that in trained males and females, RT performed with a 1–3 RIR target yields comparable gains in muscle hypertrophy and strength to reaching momentary muscular failure. These findings provide practical evidence that approaching, rather than necessarily reaching, momentary muscular failure may provide a sufficient stimulus for hypertrophy and strength adaptations. While it is possible that small, muscle-specific differences may exist, the general pattern of results supports the use of RIR-based approaches for the elbow flexors and across both sexes.

## Figures and Tables

**Table 1:** Estimates for within- and between-protocol raw changes in males and females. For the “between-protocol difference”, negative estimates favor the repetitions-in-reserve (RIR) protocol, while positive estimates favor the FAIL protocol. Probability that an estimate is in favor of a given protocol is indicated as pd, and probability that an estimate exceeds the typical error is indicated as pd > ROPE. cm = centimeter; HDI = highest density interval; pd = probability of direction; ROPE = region of practical equivalence.

Outcome Measure and Group	Estimate (cm)	95% HDI	pd (%)	pd > ROPE (%)
Male—elbow flexor thickness (n = 11)				
FAIL (within-protocol change)	0.255	0.162 to 0.349	100	100
RIR (within-protocol change)	0.219	0.124 to 0.315	100	100
Between-protocol difference (FAIL vs. RIR)	0.035	−0.075 to 0.136	76	50
Female—elbow flexor thickness (n = 8)				
FAIL (within-protocol change)	0.213	0.099 to 0.323	100	100
RIR (within-protocol change)	0.186	0.069 to 0.303	99	99
Between-protocol difference (FAIL vs. RIR)	0.027	−0.095 to 0.146	68	46

**Table 2:** Estimates for between-protocol standardized effects for the full cohort (both sexes combined) and males and females separately. The table displays the standardized effect estimates and their corresponding 95% highest density credible intervals. Negative estimates favor the repetitions in reserve (RIR) protocol, while positive estimates favor the FAIL protocol. Standardized effect estimates derived using both weakly informative priors and default (non-informative) priors. Probability that an estimate exceeds the pre-determined ROPE of −0.1 to 0.1 is indicated as pd > ROPE. HDI = highest density interval; pd = probability of direction; ROPE = region of practical equivalence.

Variable of Interest	Standardized Effect Estimate (95% HDI)	
	Default Priors	Weakly Informative Priors
Bicep thickness		
Full cohort (both sexes)	0.174 (−0.245 to 0.595): pd > ROPE = 64%	0.112 (−0.194 to 0.385): pd > ROPE = 54%
Males	0.197 (−0.366 to 0.728): pd > ROPE = 65%	0.134 (−0.229 to 0.497): pd > ROPE = 57%
Females	0.144 (−0.488 to 0.809): pd > ROPE = 56%	0.080 (−0.212 to 0.355): pd > ROPE = 44%

**Table 3:** Estimates for within- and between-protocol changes in 1RM strength for male and female participants. For the “between-protocol difference”, negative estimates favor the repetitions in reserve (RIR) protocol, while positive estimates favor the FAIL protocol. Probability that an estimate is in favor of a given protocol is indicated as pd. 1RM = 1-repetition maximum; HDI = highest density interval; kg = kilogram; pd = probability of direction.

Outcome Measure and Group	Estimates (kg)	95% HDI	pd (%)
Male elbow flexor strength (1RM)			
FAIL (within-protocol change)	7.16	5.26 to 9.18	100
RIR (within-protocol change)	6.32	4.39 to 8.43	100
Between-protocol difference (FAIL vs. RIR)	0.835	−0.661 to 2.20	88
Female elbow flexor strength (1RM)			
FAIL (within-protocol change)	4.40	1.92 to 6.88	100
RIR (within-protocol change)	4.15	1.73 to 6.63	100
Between-protocol difference (FAIL vs. RIR)	0.248	−1.308 to 1.97	62

**Table 4:** Training volume progression throughout the 8-week intervention.

Exercise	Sets per Limb per Exercise	
	Week 1–4	Week 5–8
Unilateral preacher curl	5	6

## Data Availability

The data that support the findings of this study are available from the corresponding author upon reasonable request.

## Supplementary Materials

The following supporting information can be downloaded at https://www.mdpi.com/article/10.3390/muscles5030061/s1, Section S1.1: Exercise control during the resistance training intervention; Section S1.2: Visual Demonstration of Exercise Technique; Section S1.3: Example Ultrasound Images and Reliability Scores; Figure S1: Visual demonstration of the unilateral preacher curl technique; Figure S2: Representative ultrasound images and reliability indices for elbow flexor muscle thickness; Figure S3: Raw elbow flexor thickness at pre- and post-intervention; Figure S4: Within- and between-protocol estimates for male and female elbow flexor thickness; Figure S5: Raw unilateral preacher curl 1RM strength; Figure S6: Within- and between-protocol estimates for male and female elbow flexor maximal strength.
