# Supplementary material for: Elbow Flexor Adaptations to Failure Versus Repetitions-in-Reserve Training: A Unilateral Within-Participant Study in Resistance-Trained Adults
Source: Muscles. 2026 Aug 30;5(3):61. doi: 10.3390/muscles5030061 (PMC13609929; doi:10.3390/muscles5030061)
Supplement: Supplementary file 1 [file muscles-05-00061-s001.zip › muscles-4461328-supplementary.pdf]

Elbow flexor adaptations to failure versus repetitions-in-reserve training: a unilateral within-participant study in resistance-trained adults

Supplementary Material

S1. Additional Content

S1.1 Exercise Control During Resistance Training Intervention

To minimize the effects of the experimental protocols, participants were explicitly instructed to refrain from performing any additional, unsupervised resistance training exercises that directly targeted the elbow flexors for the entire 8-week duration of the intervention. Participants were permitted to maintain their habitual training for all other, non-intervention muscle groups. Adherence to these instructions was verbally confirmed by the investigators at each supervised training session.

<table>
<tr><th>Exercise Exclusions</th></tr>
<tr><td>All direct Elbow Flexor exercises, including but not limited to:
Dumbbell, barbell, or cable bicep curls
Preacher curls (other than the study protocol)
Hammer curls</td></tr>
<tr><td>Exercise Inclusions</td></tr>
<tr><td>Permitted Training for Non-Intervention Muscle Groups
Chest: (e.g., Bench press variations, dumbbell flyes, push-ups)
Back: (e.g., straight-arm pulldowns, machine pullovers, scapular pull-ups, scapular depression/retraction drills). Exercises involving dynamic elbow flexion (e.g., rows, chin-ups, and lat pulldowns with elbow flexion) were not permitted because, despite being compound movements, they impose an additional training stimulus on the elbow flexors that could confound the experimental intervention..
Shoulders: (e.g., Overhead press variations, lateral raises, rear delt flyes)</td></tr>
</table>

S1.2 Visual Demonstration of Exercise Technique

A. Unilateral Preacher Curl

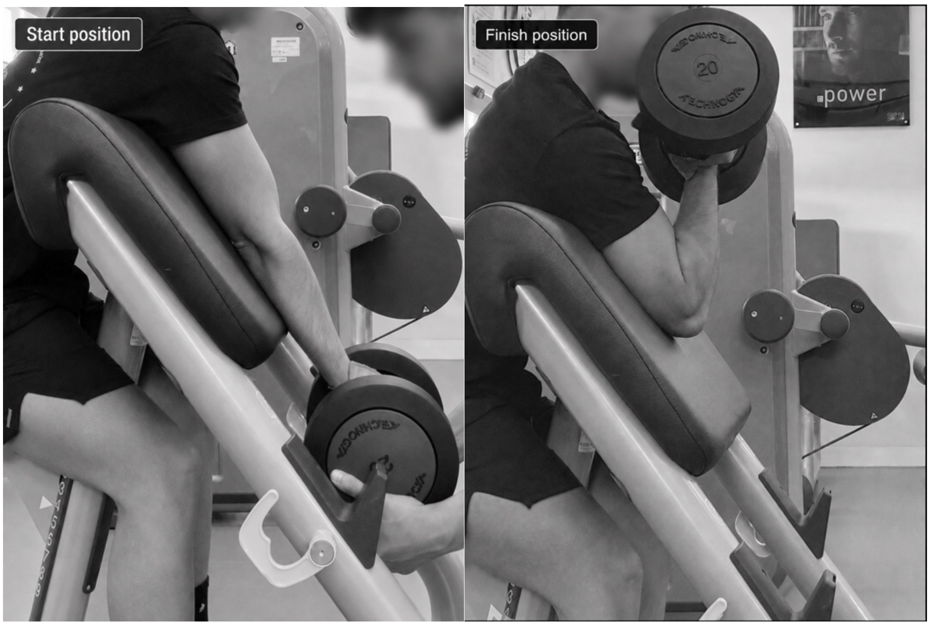

Figure S1. Visual demonstration of the unilateral preacher curl technique.

Range-of-motion was standardized for each participant during the dedicated familiarization weeks and verbally reinforced by the supervising investigator throughout the intervention to ensure proper exercise technique. Figure A shows an example of the start and end positions for the unilateral preacher curl.

S1.3 Example Ultrasound Images and Reliability Scores

Reliability of the ultrasound imaging was assessed by calculating the typical error (TE) and intraclass correlation (ICC) from the two separate assessment sessions conducted PRE and POST intervention. The typical error (TE) and intraclass correlation (ICC) from pre- and post-testing ultrasound assessments are TE = 0.035 cm and ICC = 0.997 for the elbow flexors (combination of all sites).

A. Elbow Flexors (50%, 60% and 70%, respectively)

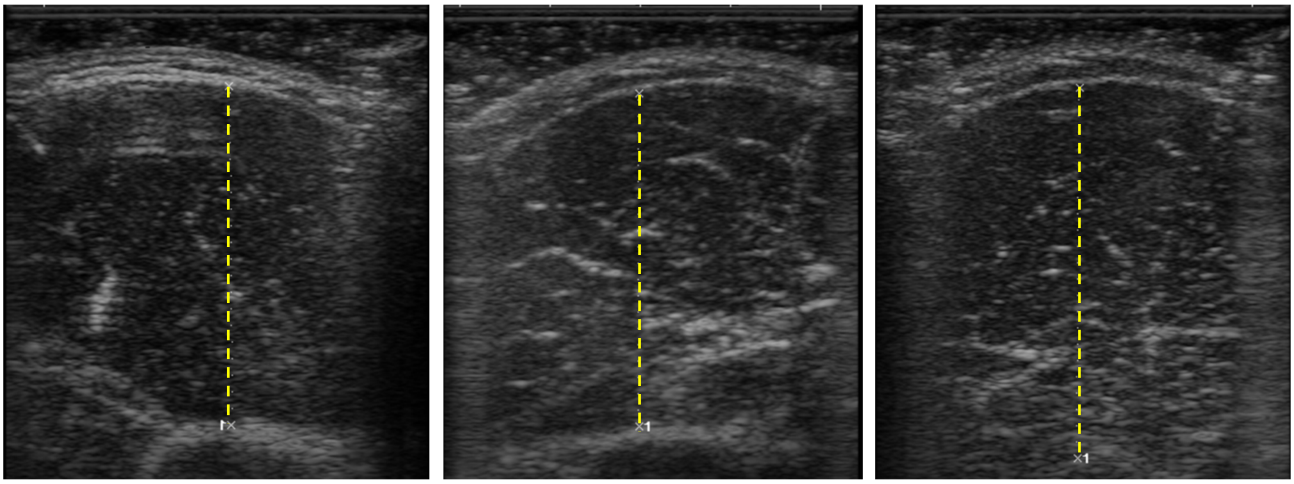

Figure S2. Representative ultrasound images and reliability indices for elbow flexor muscle thickness. *Yellow dotted line denotes sites of measurement (mm) from the superficial aponeuroses to the deep aponeuroses. The elbow flexors, the average result of three separate measurement sites (50%, 60%, and 70% of the distance from the acromion process to the cubital fossa) was used for analysis.*

S2. Additional Figures

S2.1Raw elbow flexors (50%, 60% and 70% combined) thickness at pre- and post-intervention

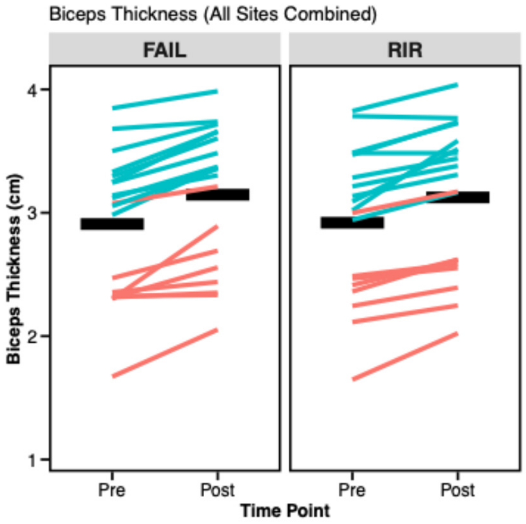

Figure S3. Raw elbow flexor thickness at pre- and post-intervention.

RIR, repetition in reserve; FAIL, Momentary muscular failure condition

S2.2 Within and between protocol estimates for males and females muscle thickness for the Elbow flexors

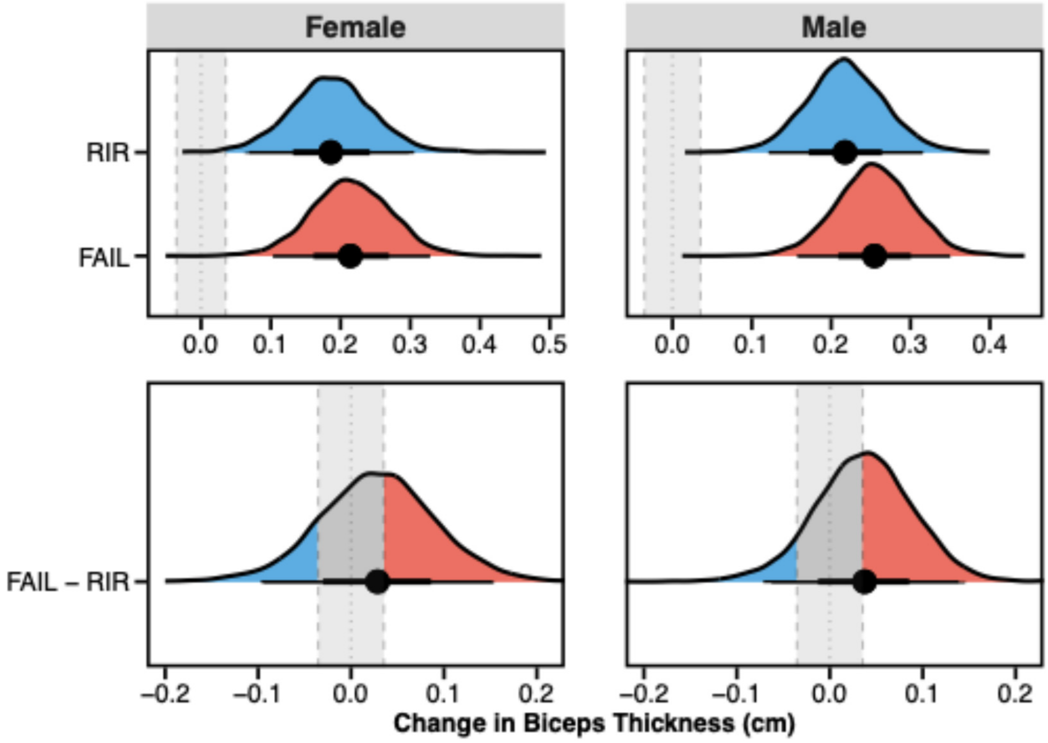

Figure S4. Within- and between-protocol estimates for male and female elbow flexor thickness.

RIR, repetition in reserve; FAIL, Momentary muscular failure condition

S2.3 Raw maximal strength for upper body

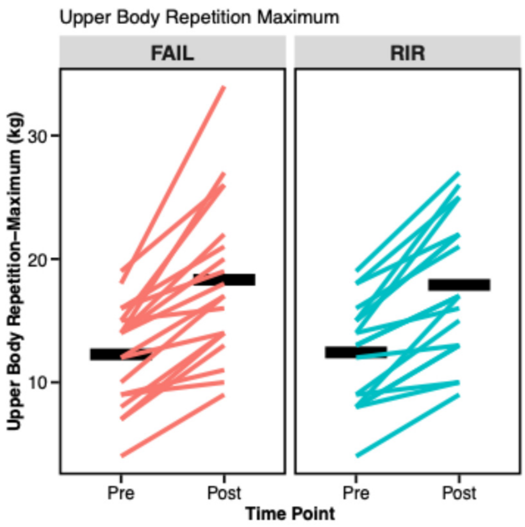

Figure S5. Raw unilateral preacher curl 1RM strength.

S2.4 Within and between protocol estimates for males and females' maximum strength for the elbow flexor

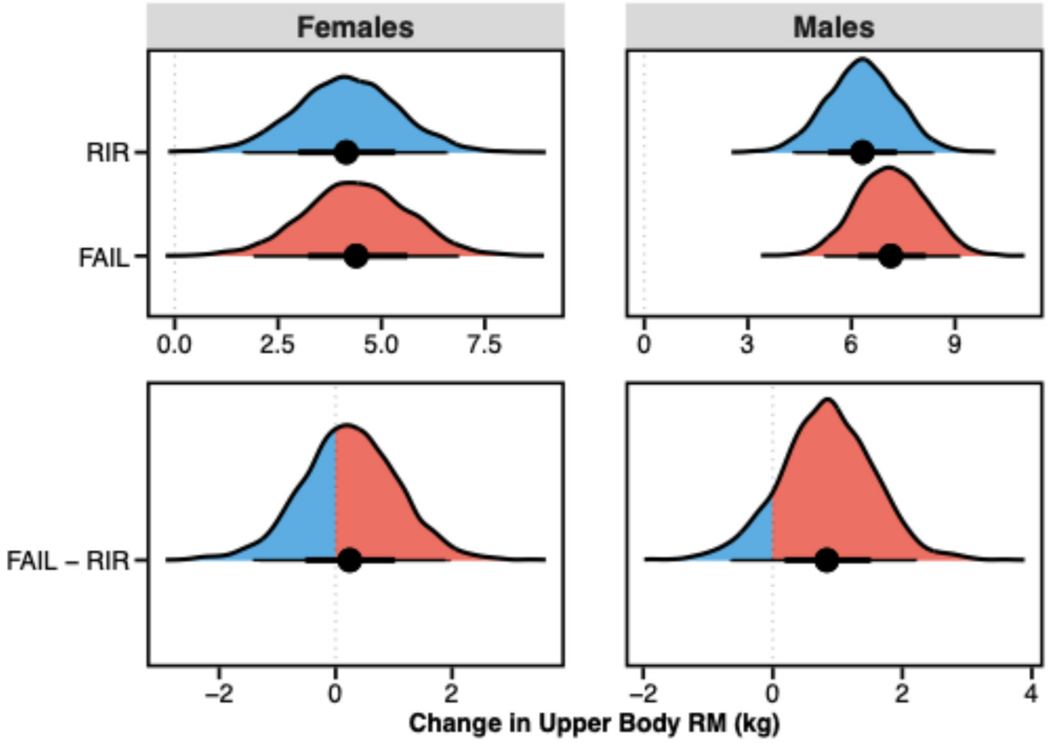

Figure S6. Within- and between-protocol estimates for male and female elbow flexor maximal strength. RIR, repetition in reserve; FAIL, Momentary muscular failure condition.
